# Natural fire-derived charcoal resembles high-temperature biochar in regulating soil CO_2_ emissions from a subtropical plantation soil

**DOI:** 10.3389/fmicb.2026.1986273

**Published:** 2026-10-29

**Authors:** Huiping Liu, Ziyue Gao, Mengyan Huang, Jiarui Man, Yao Huang, Xian Liu, Yuzhe Wang, Yalin Hu

**Affiliations:** 1 College of Forestry, Fujian Agriculture and Forestry University, Fuzhou, China; 2 National-Regional Joint Engineering Research Center for Soil Pollution Control and Remediation in South China, Guangdong Key Laboratory of Integrated Agro-environmental Pollution Control and Management, Institute of Eco-environmental and Soil Sciences, Guangdong Academy of Sciences, Guangzhou, China

**Keywords:** bacterial community, engineered biochar, natural charcoal, pyrolysis temperature, soil carbon cycling

## Abstract

Char is a carbon (C)-rich pyrogenic material generated either during natural fire events (natural charcoal) or through the thermochemical conversion of plant biomass (engineered biochar). However, whether these two types of char have comparable effects on soil CO_2_ emissions remains unclear. In this study, we conducted a 30-day incubation experiment to compare the effects of charcoal produced from *Pinus massoniana* bark during broadcast burning and engineered biochars produced from the same feedstock at 300, 500, and 900 °C (B300, B500, and B900) on soil CO_2_ emissions, physicochemical properties, and bacterial diversity and composition. The addition of B300 significantly increased soil CO_2_ emissions (*p* < 0.05), whereas B500, B900, and charcoal had no significant effects. B300 addition markedly increased the relative abundances of Proteobacteria and Cyanobacteria while decreasing those of Acidobacteriota, Chloroflexi, and Verrucomicrobiota (*p* < 0.001). Mixed-effects models accounting for treatment variation showed that soil pH was negatively associated with cumulative CO_2_ emissions, whereas Proteobacteria exhibited a positive association. Random forest analysis identified Proteobacteria and Acidobacteriota as potential predictors of cumulative CO_2_ emissions, although these relationships require further validation. In contrast, natural fire-derived charcoal caused minimal changes in bacterial community composition. Overall, our findings demonstrate that high-temperature biochars (>500 °C) exhibit similar effects on soil CO_2_ emissions and bacterial community to natural charcoal in subtropical plantation soils, highlighting their potential as functional analogues of fire-derived charcoal.

## 1 Introduction

Char is a carbon (C)-rich pyrogenic material generated through the incomplete combustion or thermochemical conversion of plant biomass. It represents an important component of terrestrial C pools and is formed through both natural and anthropogenic processes. Naturally formed charcoal is produced during wildfire events (Preston and Schmidt, 2006; Gao et al., 2024) or prescribed burning (García-Carmona et al., 2025), representing a major component of pyrogenic C inputs to soils. Owing to its chemical recalcitrance and long residence time, charcoal has been recognized as an important but previously overlooked component of the global C cycle (Santín et al., 2015), contributing to long-term atmospheric CO_2_ sequestration (Jones et al., 2019). In addition to natural fire-derived charcoal, engineered biochar has emerged as an anthropogenic form of pyrogenic C produced through the thermochemical conversion of biomass under oxygen-limited conditions (Lehmann et al., 2021; Xu and Xiong, 2025). The pyrolysis process not only transforms biomass into a more stable C form but also generates renewable energy, potentially reducing reliance on fossil fuels. Consequently, biochar has been increasingly applied as a soil amendment in agricultural and forest ecosystems to improve soil quality and enhance C sequestration (Chen et al., 2026; Mao et al., 2026; Zhou et al., 2026). Regardless of their origin, both natural charcoal and engineered biochar can regulate soil C cycling by directly contributing to soil C pools and indirectly influencing organic matter decomposition through changes in soil physicochemical properties and microbial communities.

The effects of engineered biochar on soil C sequestration and mineralization have been extensively investigated (Enebe et al., 2025; Zhang et al., 2026), whereas far fewer studies have examined the effects of natural charcoal or directly compared its influence with that of biochar. Meta-analyses have shown that the effects of biochar on soil C mineralization depend strongly on biochar properties (e.g., feedstock, pyrolysis temperature, and application rate) and site characteristics (e.g., soil texture and climate), although biochar generally stimulates soil C mineralization following its application (Chen et al., 2021; Zhang et al., 2022; Enebe et al., 2025). This stimulation has been attributed to several mechanisms, including the input of labile C, alleviation of soil acidity, and shifts in microbial community composition that increase the abundance and activity of key microbial taxa involved in C mineralization (Yang et al., 2022; Li et al., 2026). In contrast, the impacts of natural charcoal on soil C mineralization remain poorly understood. Existing evidence indicates that natural charcoal differs substantially from engineered biochar in physicochemical properties, including adsorption capacity (Pingree et al., 2016), C fractions (Howell et al., 2022), elemental composition, and chemical recalcitrance (Santín et al., 2017). For example, Santín et al. (2017) reported that wildfire-derived charcoals produced from *Pinus banksiana* wood at the highest charring temperatures exhibited lower long-term C sequestration potential than most slow-pyrolysis biochars. Whether these contrasting properties result in different effects on soil CO_2_ emissions remains largely unknown.

Soil microbial communities are key regulators of char-induced changes in soil organic C mineralization (Whitman et al., 2021; Li et al., 2026). Biochar amendment can reshape bacterial community composition by modifying key soil physicochemical properties, including pH, nutrient availability, labile C supply, and habitat structure (DeCiucies et al., 2018; Chen et al., 2021). These changes often favor copiotrophic (*r*-strategist) taxa through increased nutrient availability and labile C inputs, particularly following the application of low-temperature biochars that contain relatively large amounts of readily mineralizable C (Fang et al., 2015; Zeba et al., 2024). Conversely, oligotrophic (*K*-strategist) taxa, such as Acidobacteriota and Chloroflexi, frequently decline after biochar application, and their reduced abundance has been associated with increased soil CO_2_ emissions (Yang et al., 2022; El-Desouki et al., 2024). Although fire-derived charcoal is generally formed under higher temperatures than engineered biochar, a considerable proportion of its C has been reported to remain labile or semi-labile (Howell et al., 2022), suggesting that it may also influence soil microbial communities and C mineralization. Natural charcoal and engineered biochar differ substantially in their physicochemical properties, including C composition, aromaticity, and surface characteristics (Santín et al., 2017; Howell et al., 2022), suggesting that it may also modify soil microbial communities and C mineralization. However, it remains unclear whether natural charcoal and engineered biochar regulate soil C mineralization through similar microbial mechanisms, particularly across biochars produced at different pyrolysis temperatures.

The objective of this study was to investigate the effects of different char materials on soil CO_2_ emissions in a subtropical plantation forest. Specifically, we conducted a 30-day incubation experiment comparing natural fire-derived charcoal produced during broadcast burning and engineered biochar produced at different pyrolysis temperatures. Soil physicochemical properties and bacterial community traits were analyzed to elucidate the mechanisms underlying char-induced changes in soil CO_2_ emissions. We hypothesized that (1) charcoal would have a weaker stimulatory effect on soil CO_2_ emissions than engineered biochar because of charcoal’s lower labile C content and its limited alleviation of soil acidification, thereby resulting in weaker shifts in microbial communities associated with C mineralization; and (2) biochar produced at lower pyrolysis temperatures would promote greater soil CO_2_ emissions due to the higher retention of labile C fractions and reduced C loss during pyrolysis, thereby enhancing the abundance of microbial taxa involved in soil C mineralization. Unlike previous studies that investigated the effects of natural fire-derived charcoal on soil carbon and nutrient cycling, this study compares natural charcoal with laboratory-produced biochars generated at different pyrolysis temperatures to determine whether fire-derived charcoal shares similar functional characteristics with engineered biochars. The findings will improve our understanding of the potential use of biochar amendments in fire-affected forest ecosystems and the soil and microbial mechanisms regulating their effects on carbon cycling.

## 2 Materials and methods

### 2.1 Soil collection and char preparation

Soil used in this study was collected from a clearcut *Pinus massoniana* plantation at the Hulushan National Forest Farm in Fujian Province, southeastern China (118° E, 26° N). Broadcast burning had been conducted at the site to prevent the spread of pine wilt disease. One month after burning, surface materials (e.g., ash) were carefully removed, and mineral soil (0–10 cm depth) was collected from four sampling plots (10 × 10 m) located more than 50 m apart. Within each plot, soils from five randomly selected sampling points were composited to form one representative sample. The soil samples were transported to the laboratory in ice boxes, passed through a 2-mm sieve, thoroughly homogenized, and stored at 4 °C prior to the incubation experiment. According to the World Reference Base for Soil Resources (WRB IWG, 2015), the soil is classified as a Ferric Acrisol.

We used bark from mature *P. massoniana* to produce biochar at different pyrolysis temperatures. Air-dried bark was cut into pieces with diameters of < 1 cm and pyrolyzed at 300, 500, or 900 °C for 2 h in a tubular furnace (OTF-1200X, HF-Kejing, Hefei, China). Charcoal generated from the incomplete combustion of bark was collected in March 2021 from a clearcut *P. massoniana* stand in subjected to broadcast burning 1 month earlier (Jiao et al., 2026). The charcoal was collected around *P. massoniana* stumps, where charcoal fragments were more readily identifiable. After collection, all char materials were air-dried and stored in sealed polyethylene bags in a dry and dark environment at room temperature until the incubation experiment.

### 2.2 Laboratory incubation and gas monitoring

Four types of char amendments were applied, each with four replicates: biochar pyrolyzed at 300 °C (B300), biochar pyrolyzed at 500 °C (B500), biochar pyrolyzed at 900 °C (B900), and natural fire-derived charcoal (Charcoal). Before incubation, all char materials were oven-dried at 40 °C and ground to pass through a 2-mm sieve. Incubations were conducted in 350 mL glass jars, each containing 80 g soil on a dry mass basis and the corresponding char material at a rate of 2% (w/w). The jars were pre-incubated at 25 °C for 1 week. Thereafter, headspace gas samples were collected on days 1, 3, 5, 7, 12, 15, 18, 22, 26, and 30. Water dropwise was gradually added to maintain soil moisture at 60% of field capacity throughout the incubation. For each sampling event, 10 mL of headspace gas was extracted from the flask using a 10 mL gas-tight syringe and analyzed using a gas chromatograph (GC-2014, Shimadzu, Japan). CO_2_ emission rate (*F*, mg·kg^−1^·h^−1^) and cumulative CO_2_ emissions (C, mg·kg^−1^) were calculated using the following Equations 1, 2:


$$F=\rho \times \frac{V}{W}\times \frac{\mathrm{\Delta c}}{\mathrm{\Delta t}}\times \frac{273}{273+T}$$ (1)

Where *ρ* is the standard density of CO_2_; *V* is the headspace volume of the flask (m^3^); *W* is the dry soil mass (kg); Δ*c* is the difference in CO_2_ concentration measured over the 1 h static period (Δ*t* = 1 h); T denotes incubation temperature (25 °C); the term 273/(273 + *T*) corrects gas volume to standard temperature.


$$C=\sum_{i=1}^{n-1}\frac{F_{h,i}+F_{h,i+1}}{2}\times D_{i}\times 24$$ (2)

Where *F*_h, i_ and *F*_h, i + 1_ represent hourly CO_2_ emission rates at the *i*-th and (*i* + 1)-th sampling, respectively; *D*_i_ is the interval days between two consecutive samplings; the multiplier 24 converts hourly flux to daily emission for integral calculation.

Soil samples collected at the end of the incubation were analyzed for physiochemical and biological properties. Subsamples were stored at −80 °C prior to microbial analysis. Other subsamples were air-dried for the determination of soil pH, total C and N contents, whereas fresh soils were used for analyses of labile C and N pools.

### 2.3 Char characterization

Both biochar and charcoal were characterized for their physicochemical properties. pH was measured using a pH meter (PHS-3C, Leici, China) at a solid-to-deionized water ratio of 1:10 (w/v). Total C, N, and H contents were determined by dry combustion using an elemental analyzer (Vario EL Cube, Elementar, Germany). Oxygen (O) content was analyzed using an elemental analyzer (Vario MICRO, Elementar, Germany). Specific surface area, total pore volume, and pore size distribution were determined from N₂ adsorption–desorption isotherms measured at 77 K using a Quadrasorb S1 surface area analyzer (Quantachrome Instruments, USA).

### 2.4 Soil analysis

Soil pH was determined using a pH meter (PHS-3C, Leici, China) at a soil to deionized water ratio of 1:2.5 (w/v). Total C was measured using an elemental analyzer (vario ISOTOPE cube, Elementar, Germany) after samples were ground using a ball mill. Dissolved organic carbon (DOC) were extracted with 2 mol L^−1^ KCl solution and then measured using a total organic carbon analyzer (TOC-LCPH, Shimadzu, Japan). Microbial biomass C (MBC) and N (MBN) were determined using the chloroform fumigation-extraction method. Soils were extracted with 0.5 mol L^−1^ K_2_SO_4_ solution, and the extracts were analyzed using a TOC analyzer (TOC-LCPH, Shimadzu, Japan). Conversion coefficients of 0.45 and 0.54 were used for MBC and MBN, respectively.

Soil DNA extraction, PCR amplification, and Illumina sequencing were conducted by Beijing Allwegene Gene Technology Co., Ltd. (Beijing, China). Genomic DNA was extracted from 0.5 g frozen soils using the FastDNA® SPIN Kit for Soil (MP Biomedicals, USA), and DNA integrity was assessed by 1% agarose gel electrophoresis. Amplicons were purified, quantified, and used for library construction, then sequenced on an Illumina NovaSeq PE300 platform. Raw reads were merged with PEAR v0.9.11, quality-filtered, and chimera-checked with VSEARCH v2.13.3. Clean sequences were clustered into OTUs at 97% similarity using UPARSE (QIIME v1.8.0), rarefied to equal depth, and taxonomically assigned against SILVA 138 using the RDP Classifier (70% confidence). Relative abundances were summarized at phylum and genus levels for downstream analyses.

### 2.5 Statistical analysis

All statistical analyses were performed using R software (version 4.4.1). One-way analysis of variance (ANOVA) followed by Tukey’s HSD post-hoc test was employed to assess differences in char properties, average CO_2_ emission rate, cumulative CO_2_ emission, soil physicochemical properties, bacterial alpha-diversity indices, and taxon relative abundances. *β*-diversity was evaluated via non-metric multidimensional scaling (NMDS) based on Bray–Curtis dissimilarity matrices, with significant group discrimination determined by permutational multivariate analysis of variance (PERMANOVA). Linear mixed-effects models were performed to evaluate the associations of soil properties and bacterial taxa with cumulative CO_2_ emissions while accounting for treatment-level variation. Cumulative CO_2_ emissions were set as the response variable, each soil property and bacterial taxon was included separately as a fixed-effects predictor, and treatment was included as a random intercept. To identify the potential predictors of CO_2_ emissions, Random Forest regression was implemented, and variable importance was ranked using the percent increase in mean squared error (%IncMSE). Statistical significance was determined using a two-tailed threshold of *p* < 0.05 for all statistical tests.

## 3 Results

### 3.1 Characteristics of engineered biochar and natural charcoal

With increasing pyrolysis temperature from 300 to 900 °C, char C content, N content, C/H ratio, specific surface area, and total pore volume increased progressively, whereas H content, O content, and C/N ratio decreased markedly (Table 1 and Supplementary Table 1, *p* < 0.001). The natural charcoal had a significantly higher N content but lower C content and C/N ratio than biochar (*p* < 0.001). The H and O contents of charcoal were significantly higher than those of B900 but lower than those of B300 and B500 (*p* < 0.001). Regarding pore structure, the natural charcoal displayed significantly greater surface area and total pore volume than B300 and B500, yet remained substantially lower than B900 (*p* < 0.001). Charcoal had a significantly larger pore size than B300 and B900, but smaller than that of B500 (*p* < 0.001).

**Table 1:** Characteristics of engineered biochar and natural charcoal (mean ± SE, *n* = 3).

Property	B300	B500	B900	Charcoal
C (%)	60.60 ± 0.10c	72.50 ± 0.10b	86.50 ± 0.05a	48.90 ± 0.03d
H (%)	6.14 ± 0.08a	4.33 ± 0.02b	1.58 ± 0.05d	3.36 ± 0.09c
O (%)	28.50 ± 0.10a	18.60 ± 0.19b	4.70 ± 0.16d	16.50 ± 0.06c
N (%)	0.44 ± 0.05d	0.57 ± 0.02c	0.79 ± 0.05b	1.41 ± 0.09a
C/H	9.88 ± 0.12c	16.76 ± 0.07b	54.75 ± 1.66a	14.57 ± 0.40b
C/N	139.4 ± 8.0a	127.7 ± 1.6a	110.2 ± 4.0b	34.7 ± 1.2c
Surface area (m^2^ g^−1^)	1.14 ± 0.42c	1.49 ± 0.03c	327.30 ± 3.40a	10.10 ± 0.11b
Pore volume (cc kg^−1^)	2.90 ± 0.50d	4.10 ± 0.20c	154.50 ± 0.10a	12.40 ± 0.30b
Pore size (Å)	25.20 ± 4.40c	110.00 ± 7.60a	18.90 ± 0.20c	49.20 ± 1.20b

Different letters within the same row indicate significant differences among treatments (*p* < 0.05). B300, B500, and B900 denote biochars pyrolyzed at 300, 500, and 900 °C, respectively.

### 3.2 Soil CO_2_ emission

Soil CO_2_ emission rates exhibited distinct temporal patterns among treatments during the incubation period (Figure 1a). B300 markedly stimulated CO_2_ emissions during the early incubation stage compared with the Control, B500, B900, and the Charcoal treatments (Figures 1a,b). Accordingly, both the average CO_2_ emission rate and cumulative CO_2_ emissions were significantly higher in the B300 treatment than in the Control (Figures 1c,d, *p* < 0.05), whereas B500, B900, and Charcoal had no significant effects on either parameter (*p* > 0.05).

**Figure 1:**
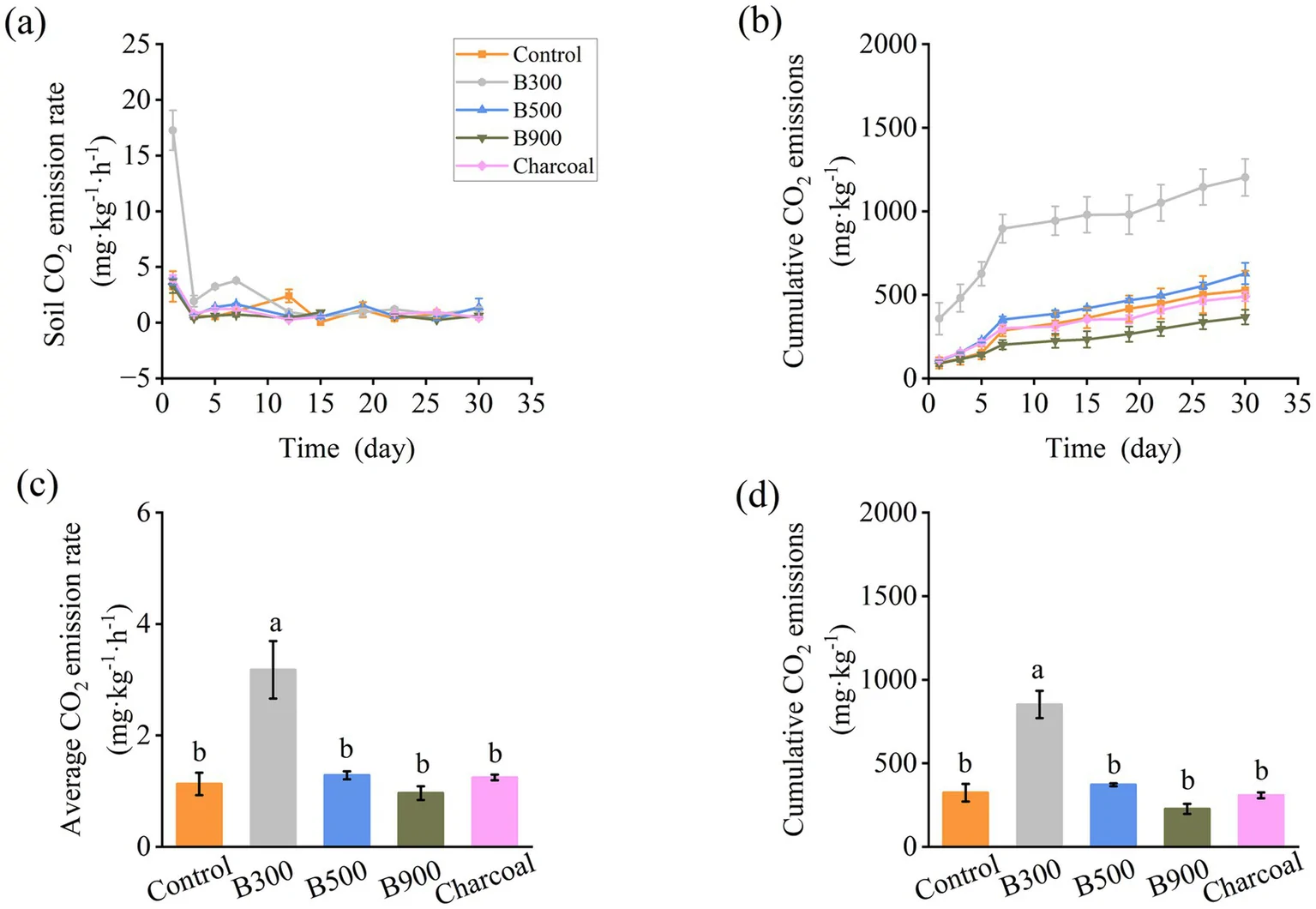
Soil CO_2_ emission dynamics under varying char addition treatments. **(a)** Dynamics of the CO_2_ emission rate, **(b)** Dynamics of cumulative CO_2_ emissions, **(c)** Average CO_2_ emission rate, and **(d)** Cumulative CO_2_ emissions. Different lowercase letters indicate significant differences among treatments (*p* < 0.05). B300, B500, and B900 denote biochars pyrolyzed at 300, 500, and 900 °C, respectively.

### 3.3 Soil physiochemical properties

The application of biochar and charcoal significantly altered several soil chemical properties (Table 2 and Supplementary Table 2). Soil pH increased significantly in all amended treatments relative to the Control (*p* < 0.001), with the highest value observed in B900, whereas B300, B500, and Charcoal exhibited similar intermediate values. Total C content was also significantly increased by all amendments (*p* < 0.001), with B900 resulting in the highest total C concentration. Soil DOC content was significantly lower in the B500, B900, and Charcoal treatments than in the Control (*p* < 0.001), whereas no significant difference was found between the Control and B300. Soil DOC content in the B300 treatment was significantly higher than that in the B500 and B900 treatments, which were in turn significantly higher than that in the Charcoal treatment (*p* < 0.001). No significant differences in MBC or MBN were detected among the treatments (*p* > 0.05).

**Table 2:** Soil properties at the end of the incubation period (mean ± SE, *n* = 4).

Treatment	pH	TC (g kg^−1^)	DOC (mg kg^−1^)	MBC (mg kg^−1^)	MBN (mg kg^−1^)
Control	4.47 ± 0.01c	13.0 ± 0.6c	89.3 ± 5.0a	540.0 ± 61.4a	276.6 ± 9.9a
B300	4.96 ± 0.13b	37.6 ± 2.2b	81.9 ± 4.7a	842.2 ± 149.8a	153.4 ± 41.2a
B500	4.96 ± 0.02b	39.8 ± 2.5b	78.8 ± 6.0b	576.8 ± 152.1a	369.2 ± 108a
B900	5.34 ± 0.04a	46.5 ± 1.8a	75.6 ± 2.6b	500.2 ± 61.9a	205.2 ± 35.8a
Charcoal	4.96 ± 0.02b	36.9 ± 1.4b	60.6 ± 1.8c	751.4 ± 148.1a	284.4 ± 63.7a

Different letters within the same column indicate significant differences among treatments (*p* < 0.05). TC, total carbon; DOC, dissolved organic C; MBC, microbial biomass C; MBN, microbial biomass nitrogen. B300, B500, and B900 denote biochars pyrolyzed at 300, 500, and 900 °C, respectively.

### 3.4 Soil bacterial community diversity and composition

The OTU richness, Shannon diversity index, and Simpson diversity index were significantly higher in the B500, B900, and Charcoal treatments than in the Control and B300 treatments (Table 3, *p* < 0.001). Among all treatments, B900 exhibited the highest OTU richness and Shannon diversity. The Chao 1 index was significantly higher in the B900 treatment than in the Control (*p* < 0.001), whereas the B300, B500, and Charcoal treatments did not differ significantly from the Control (*p* > 0.05).

**Table 3:** Alpha diversity indices of soil bacterial communities at the end of the incubation period (mean ± SE, *n* = 4).

Treatment	OTUs	Shannon index	Simpson index	Chao1
Control	2,183 ± 10c	9.04 ± 0.01c	0.99 ± 0.00b	3,091 ± 49b
B300	2,157 ± 15c	9.00 ± 0.00c	0.99 ± 0.00b	3,131 ± 47ab
B500	2,307 ± 14b	9.19 ± 0.02b	1.00 ± 0.00a	3,237 ± 26ab
B900	2,423 ± 20a	9.27 ± 0.02a	1.00 ± 0.00a	3,308 ± 74a
Charcoal	2,307 ± 11b	9.18 ± 0.01b	1.00 ± 0.00a	3,264 ± 54ab

Different letters within the same column indicate significant differences among treatments (*p* < 0.05). B300, B500, and B900 denote biochars pyrolyzed at 300, 500, and 900 °C, respectively.

The dominant bacterial phyla across all treatments were Acidobacteriota, Proteobacteria, Chloroflexi, and Verrucomicrobiota (Figure 2a). NMDS ordination revealed significant differences in soil bacterial community composition among treatments (Stress = 0.076, Figure 2b). The B300 treatment was clearly separated from the other treatments, whereas B500 was positioned relatively clustered close to the Control. The B500, B900, and Charcoal treatments occupied relatively close positions in the ordination space. Although pairwise PERMANOVA showed significant differences among all treatment pairs (*p* < 0.05, Supplementary Table 3), the differences between charcoal and the high-temperature biochars (B500 and B900) were smaller than that between Charcoal and B300, as indicated by the lower F and R^2^ values for B500 vs. Charcoal (*F* = 3.33, *R*^2^ = 0.36) and B900 vs. Charcoal (*F* = 3.81, *R*^2^ = 0.39) than for B300 vs. Charcoal (*F* = 10.2, *R*^2^ = 0.63).

**Figure 2:**
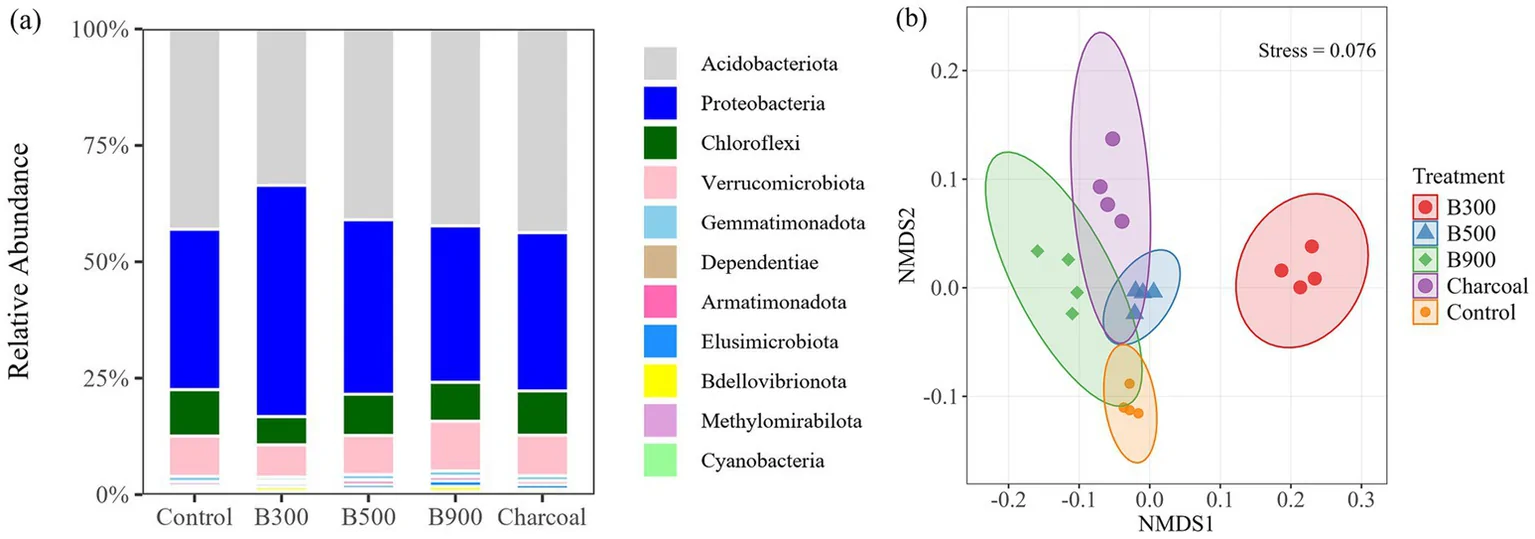
Effects of engineered biochar and natural charcoal on soil bacterial community composition. **(a)** Relative abundance of dominant bacterial phyla; **(b)** Non-metric multidimensional scaling (NMDS) ordination of community structures based on OTU data (stress = 0.076). B300, B500, and B900 denote biochars pyrolyzed at 300, 500, and 900 °C, respectively.

Biochar application significantly altered the relative abundances of several soil bacterial phyla (Figure 3). Compared with the Control and Charcoal treatments, all biochar treatments (B300, B500, and B900) exhibited significantly lower abundances of Acidobacteriota and Chloroflexi (*p* < 0.001), with the lowest abundance observed in the B300 treatment. The relative abundance of Proteobacteria was significantly higher in the B300 treatment than in the B500 treatment, whereas both treatments exhibited significantly higher abundances than the Control, B900, and Charcoal treatments, which did not differ significantly from one another (*p* < 0.001). Verrucomicrobiota was significantly less abundant in the B300 treatment but more abundant in the B900 treatment compared with the Control (*p* < 0.001), whereas no significant differences were observed among the B500, Charcoal, and Control treatments (*p* > 0.05). In contrast, Cyanobacteria was significantly more abundant in all biochar treatments than in the Control (*p* < 0.001), with the highest abundance observed in the B300 treatment. Gemmatimonadota was significantly less abundant only in the B300 treatment than in all other treatments (*p* < 0.001, Supplementary Table 4), whereas Armatimonadota was significantly less abundant in the B300 treatment but more abundant in the B500 treatment than in the Control (*p* < 0.001). The relative abundances of soil bacterial taxa at the genus level were summarized in Supplementary Table 5.

**Figure 3:**
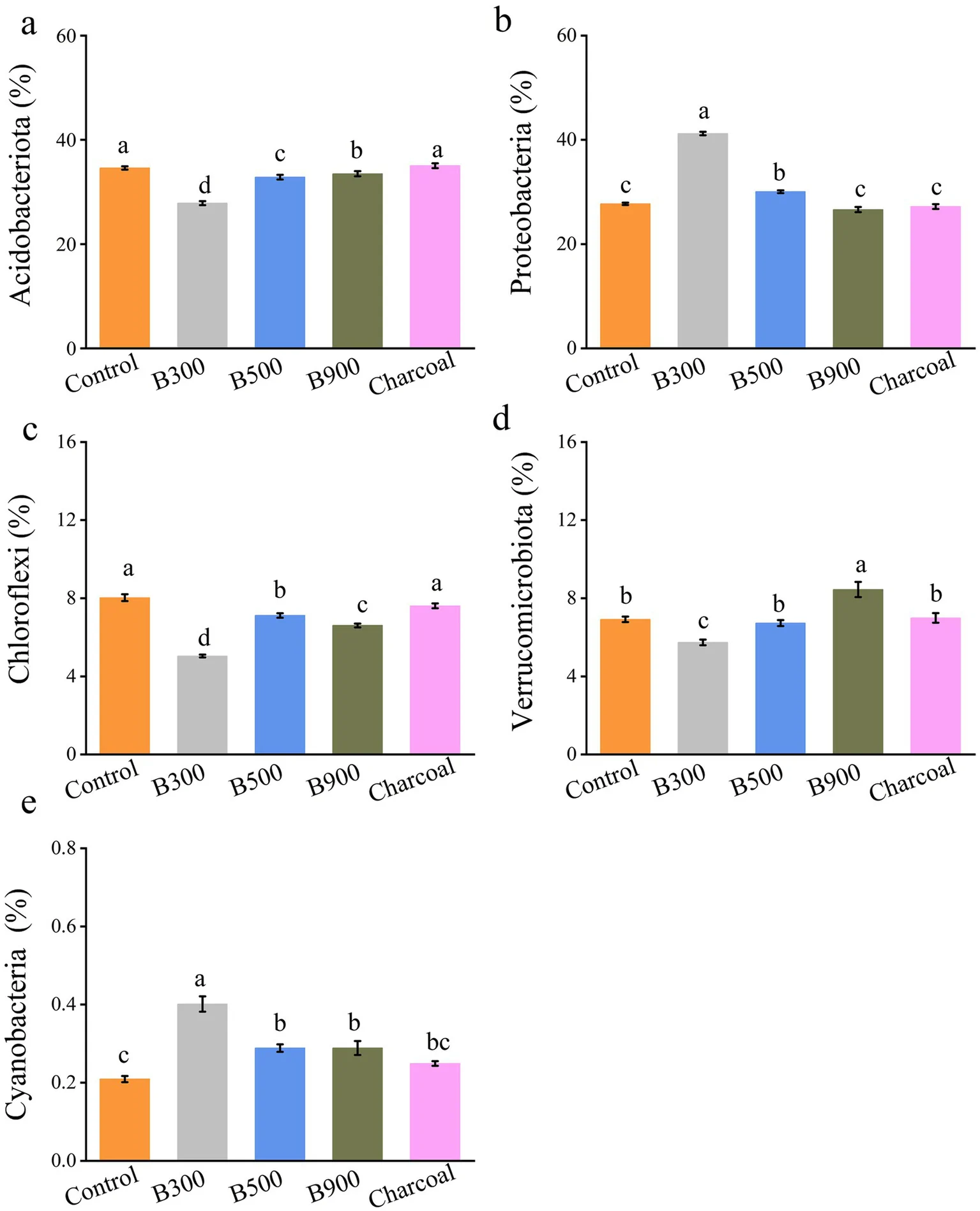
Relative abundances of key bacterial phyla across treatments: **(a)** Acidobacteriota, **(b)** Proteobacteria, **(c)** Chloroflexi, **(d)** Verrucomicrobiota, and **(e)** Cyanobacteria. B300, B500, and B900 denote biochars pyrolyzed at 300, 500, and 900 °C, respectively.

### 3.5 Relationships between CO_2_ emission and soil properties

Soil pH showed a negative association with cumulative CO_2_ emissions, whereas Proteobacteria exhibited a positive association after accounting for treatment effects (Table 4). In contrast, DOC and other bacterial phyla were not significantly associated with cumulative CO_2_ emissions. The Random Forest regression model explained a substantial proportion of the variation in cumulative soil CO_2_ emissions (*R*^2^ = 0.745, *p* < 0.001; Figure 4). Variable importance analysis based on the percentage increase in mean squared error (%IncMSE) identified Proteobacteria as the most influential predictor, followed by Acidobacteriota, Chloroflexi, Verrucomicrobiota, and Cyanobacteria. Among soil physicochemical variables, DOC and pH were also identified as important predictors, although their contributions were weaker than those of the dominant bacterial phyla.

**Table 4:** Associations of soil properties and bacterial taxa with cumulative CO_2_ emissions based on linear mixed-effects models accounting for char treatment variation.

Soil property	Estimate (SE)	*t*	*df*	*p*	Standardized *beta*
pH	−405.3 (144.9)	−2.80	16.8	0.018*	−0.51
DOC	−1.8 (3.0)	−0.62	16.3	0.564	−0.11
Acidobacteriota	−39.0 (20.8)	−1.87	8.8	0.148	−0.44
Proteobacteria	39.2 (4.2)	9.46	3.1	0.002**	0.91
Chloroflexi	−43.3 (71.4)	−0.61	9.6	0.619	−0.19
Verrucomicrobiota	−33.7 (48.8)	−0.69	17.8	0.537	−0.14
Cyanobacteria	−252.4 (820.7)	−0.31	17.9	0.784	−0.07

The estimate represents the change in cumulative CO_2_ emissions (mg CO_2_ kg^−1^ soil) per unit increase in the predictor variable; SE, standard error; df, Kenward-Roger denominator degrees of freedom. Standardized *β* was obtained after *z*-standardizing both the response and predictor. *p < 0.05, **p < 0.01.

**Figure 4:**
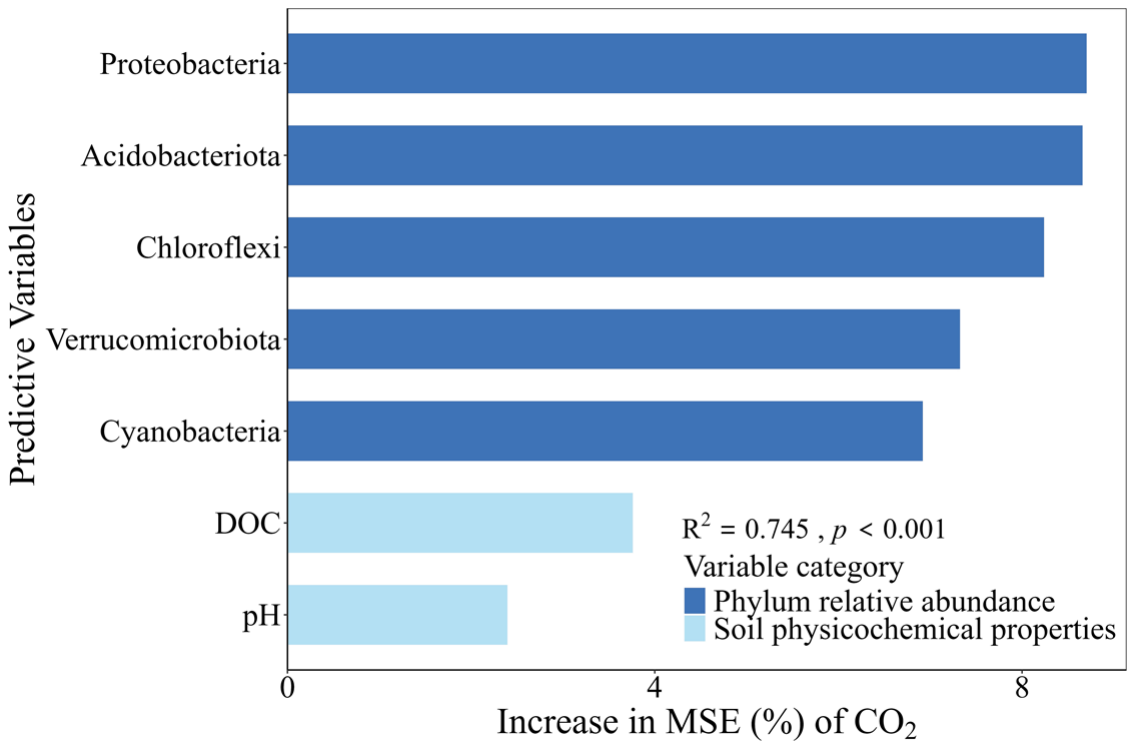
Random forest variable importance (%IncMSE) for cumulative CO_2_ emissions. Blue bars indicate bacterial phyla relative abundance, and Azure bars indicate soil physicochemical properties. The random forest model explained 74.5% of the variation in cumulative CO_2_ emissions (*R*^2^ = 0.745, *p* < 0.001).

## 4 Discussion

### 4.1 Effects of engineered biochar and natural charcoal on soil CO_2_ emissions

Soil CO_2_ emissions responded differently to biochar produced at different pyrolysis temperatures, with low-temperature biochar exerting the significant stimulatory effect. In the present study, both the average CO_2_ emission rate and cumulative CO_2_ emissions were significantly higher in the B300 treatment than in the B500 and B900 treatments (Figures 1c,d), consistent with previous studies (Figueiredo et al., 2019; Yang et al., 2022). For example, Yang et al. (2022) reported that maize-straw biochar produced at 300 °C significantly increased cumulative CO_2_ emissions from a sandy loam soil, whereas biochar produced at 450 and 600 °C had no significant effect. This pyrolysis temperature dependence has generally been attributed to the higher content of labile organic C compounds in low-temperature biochar (Zhao et al., 2018), together with its greater capacity to stimulate microbial activity and alter bacterial community composition (Yang et al., 2022). Although low-temperature biochar often increases soil pH more effectively than high-temperature biochar (Yuan et al., 2011), soil pH was lower in the B300 treatment than in the B900 treatment in our study, suggesting that pH was not associated with the enhanced CO_2_ emissions. Instead, the relatively high DOC concentration in the B300 treatment (Table 2), which was significantly higher than those in the B500, B900, and Charcoal treatments, suggests greater availability of potentially labile C under B300. This greater C availability may have contributed to the higher microbial activity and shifts in bacterial community composition observed in B300, potentially facilitating soil organic C mineralization and CO_2_ production. However, because DOC was not significantly different between B300 and the Control, and because our experimental design did not directly establish a causal relationship between DOC availability and microbial responses, these mechanisms should be interpreted as potential rather than demonstrated causal pathways. The microbial patterns potentially underlying these responses are discussed in the following section.

Natural fire-derived charcoal exhibited a markedly different effect on soil CO_2_ emissions from low-temperature biochar but behaved similarly to biochar produced at higher pyrolysis temperatures. Cumulative CO_2_ emissions in the Charcoal treatment were comparable to those in the B500 and B900 treatments but were significantly lower than those in the B300 treatment (Figure 1d). This difference may be associated with the lower availability of labile C following charcoal addition, as indicated by the lower soil DOC content and the absence of a significant increase in the relative abundance of copiotrophic bacteria such as Proteobacteria at the end of the incubation (Table 2; Figure 3b). Compared with engineered biochar produced at 300 °C, natural charcoal is generally exposed to higher combustion temperatures, resulting in greater aromatic condensation and lower concentrations of readily decomposable organic compounds (Keiluweit et al., 2010). Consequently, charcoal provides fewer substrates for microbial metabolism and has a weaker stimulatory effect on soil C mineralization. Although the carbon sequestration potential of fire-derived charcoal and engineered biochar has been compared previously (Santín et al., 2017), direct comparisons of their effects on soil organic C mineralization remain scarce. Our results demonstrate that natural charcoal and high-temperature biochar (> 500 °C) exert comparable effects on soil CO_2_ emissions, suggesting that the stability of char materials, rather than their origin, is a key determinant of their influence on soil C mineralization.

### 4.2 Microbial mechanisms underlying char-induced changes in soil CO_2_ emissions

The effects of char addition on soil CO_2_ emissions were closely associated with shifts in copiotrophic bacterial taxa, particularly Proteobacteria, which generally exhibit an *r*-strategist lifestyle and rapidly respond to increases in labile C availability (Fierer et al., 2007). In this study, cumulative soil CO_2_ emissions were positively correlated with Proteobacteria abundance after accounting for char treatment variation (Table 4), and the enhanced CO_2_ emissions under the B300 treatment coincided with a significant increase in Proteobacteria abundance (Figures 2d, 3b). The higher DOC contents observed under B300 at the end of the incubation (Table 2) may indicate greater availability of labile C, potentially favoring copiotrophic bacteria. This interpretation is consistent with previous studies showing that low-temperature biochar can stimulate microbial activity by providing readily available C substrates and nutrients (Yang et al., 2022; Cao et al., 2025). However, because DOC and bacterial communities were measured only at the end of the incubation, these observations represent associations rather than evidence of a temporal causal mechanism. Random forest analysis also identified Proteobacteria abundance as a potential predictor of cumulative soil CO_2_ emissions, although this exploratory relationship should be interpreted cautiously given the limited number of experimental replicates.

In addition to promoting copiotrophic taxa, char addition altered the relative abundances of oligotrophic bacterial groups such as Acidobacteria and Chloroflexi, which are commonly associated with nutrient-poor environments (Kalam et al., 2020; Zhang et al., 2026). Acidobacteria dominated the bacterial community in our acidic soils, consistent with previous observations in similar ecosystems (Wang et al., 2020, 2022). Biochar addition significantly reduced the relative abundances of Acidobacteria and Chloroflexi, with the strongest suppression occurring in the B300 treatment, which also produced the greatest increase in cumulative CO_2_ emissions (Figures 1d, 3a). As an oligotrophic bacterial group adapted to nutrient-poor environments, Chloroflexi are generally less competitive under conditions of increased labile C availability (Song et al., 2019; El-Desouki et al., 2024). The higher DOC concentration in the B300 treatment at the end of incubation may have favored copiotrophic taxa such as Proteobacteria over oligotrophic groups. However, after accounting for treatment-level variation, neither Acidobacteria nor Chloroflexi showed a significant independent association with cumulative CO_2_ emissions (Table 4). Thus, the observed shifts in these bacterial groups may reflect broader changes in bacterial community composition following char addition rather than direct drivers of the CO_2_ response.

### 4.3 Implications for biochar application and soil carbon management

Natural fire-derived charcoal is produced during both wildfires and prescribed burning (Preston and Schmidt, 2006; García-Carmona et al., 2025). In subtropical plantations, burning of harvest residues remains a common management practice for site preparation and reducing the risk of plant diseases (Guo et al., 2006; Huang et al., 2013). Our previous studies have demonstrated that charcoal deposited during broadcast burning in *P. massoniana* plantations contributes to post-fire soil recovery by promoting microbial biomass (Jiao et al., 2024), improving soil water retention (Cheng et al., 2024), and enhancing soil organic C stabilization (Wang et al., 2025). Consistent with these findings, the present incubation experiment showed that fire-derived charcoal did not stimulate soil CO_2_ emissions, suggesting its potential to facilitate post-fire soil recovery while minimizing soil C losses through mineralization. However, because this study was conducted under controlled laboratory conditions, future long-term field studies are needed to verify whether the observed responses persist under natural environmental conditions.

From a management perspective, charcoal inputs may be enhanced through two complementary approaches. First, charcoal production during residue burning can be increased by optimizing combustion conditions, such as fuel moisture, fuel composition, and burning intensity, to favor incomplete combustion and char formation. Second, where natural charcoal production is limited, engineered biochar may serve as an effective alternative. Our results showed that biochar produced at higher pyrolysis temperatures (500 and 900 °C) exerted effects on soil CO_2_ emissions comparable to those of natural fire-derived charcoal, whereas low-temperature biochar (300 °C) substantially stimulated soil C mineralization. Therefore, high-temperature biochar is likely to be a more suitable amendment for subtropical plantations managed with broadcast burning, as it can mimic the ecological functions of natural fire-derived charcoal while minimizing additional soil CO_2_ emissions. These findings provide a practical basis for selecting biochar with appropriate production temperatures to support sustainable soil carbon management in fire-managed forest ecosystems.

### 4.4 Limitations and future perspectives

Although this study provides new insights into the effects of natural fire-derived charcoal and engineered biochars on soil CO_2_ emissions and microbial communities, several limitations should be acknowledged, and future research is needed to further understand their long-term ecological implications. First, although all char materials originated from *P. massoniana* bark, natural charcoal was collected from a single field fire event, whereas engineered biochars were produced under controlled pyrolysis conditions. Therefore, differences among char types may reflect not only pyrolysis temperature but also variations in combustion conditions and other formation processes. Future studies using natural charcoal from multiple fire events and feedstock types are needed to better characterize the variability of fire-derived charcoal. Second, the 30-day incubation experiment mainly reflects short-term microbial respiration responses and may not represent long-term changes in soil carbon stabilization, microbial adaptation, and carbon turnover occurring over seasonal or inter-annual timescales. Long-term field experiments are therefore needed to evaluate the persistence and ecological consequences of charcoal and biochar inputs. Third, our microbial analyses focused on bacterial communities, whereas fungi play critical roles in organic matter decomposition and soil carbon cycling. Integrating fungal community analyses and functional measurements in future studies would provide a more comprehensive understanding of microbial mechanisms underlying char-induced changes in soil respiration. Finally, the correlation and random forest analyses linking soil properties and microbial taxa with CO_2_ emissions should be interpreted cautiously due to the limited number of replicates and the lack of temporal measurements, as soil properties and microbial communities were characterized only at the end of incubation. Future studies with larger datasets, repeated temporal sampling, and independent validation are needed to confirm the robustness of identified predictors and better elucidate the mechanisms regulating CO_2_ emissions following charcoal and biochar additions.

## 5 Conclusion

In this study, we compared the effects of natural fire-derived charcoal and engineered biochar produced at different pyrolysis temperatures on soil CO_2_ emissions and bacterial community composition in a subtropical plantation soil. Biochar produced at 300 °C significantly increased soil CO_2_ emissions, whereas fire-derived charcoal and biochar produced at 500 and 900 °C had no significant effect. The stimulatory effect of low-temperature (300 °C) biochar was accompanied by increased relative abundances of Proteobacteria and Cyanobacteria and decreased abundances of Acidobacteriota, Chloroflexi, and Verrucomicrobiota. Mixed-effects analyses further showed that soil pH was negatively associated with cumulative CO_2_ emissions, whereas Proteobacteria showed a positive association after accounting for treatment-level variation. In contrast, natural fire-derived charcoal did not substantially alter bacterial community composition or stimulate soil CO_2_ emissions. Overall, our findings demonstrate that the effects of engineered biochar on soil CO_2_ emissions depend strongly on pyrolysis temperature, with high-temperature biochars showing responses similar to natural fire-derived charcoal in this subtropical plantation soil. These findings provide insights into the potential use of high-temperature biochar as a functional analogue of fire-derived charcoal, although longer-term experiments and studies across a broader range of fire events, production conditions, and forest soils are needed to evaluate its applicability to fire-affected forest ecosystems.

## Data Availability

The raw sequence data generated in this study have been deposited in the NCBI Sequence Read Archive under BioProject accession number PRJNA1535798. All other datasets supporting the conclusions of this article are available from the corresponding author upon reasonable request.
