# Supplementary material for: Natural fire-derived charcoal resembles high-temperature biochar in regulating soil CO2 emissions from a subtropical plantation soil
Source: Front Microbiol. 2026 Oct 29;17:1986273. doi: 10.3389/fmicb.2026.1986273 (PMC13755320; doi:10.3389/fmicb.2026.1986273)
Supplement: Supplementary file 1 [file Table_1.DOCX]

**Supplementary** **TABLE S1** One-way ANOVA results for properties of charcoal and biochars produced at different pyrolysis temperatures.

| Property | *F* | *p* |
| --- | --- | --- |
| C | 44747.2 | < 0.001 |
| H | 849.0 | < 0.001 |
| O | 5273.7 | < 0.001 |
| N | 182.5 | < 0.001 |
| C/N | 104.7 | < 0.001 |
| C/H | 582.6 | < 0.001 |
| Surface Area | 8899.3 | < 0.001 |
| Pore Volume | 64877.1 | < 0.001 |
| Pore Size | 89.5 | < 0.001 |

**Supplementary TABLE S2** One-way ANOVA results for soil chemical properties and bacterial community characteristics under different char addition treatments.

| Property | *F* | *p* |
| --- | --- | --- |
| pH | 22.876 | < 0.001 |
| TC | 47.229 | < 0.001 |
| DOC | 11.366 | < 0.001 |
| MBC | 1.958 | 0.153 |
| MBN | 89.5 | 0.573 |
| OTUs | 55.199 | < 0.001 |
| Shannon index | 62.517 | < 0.001 |
| Simpson index | 9.735 × 10^28^ | < 0.001 |
| Chao1 | 3.044 | 0.051 |

TC, total carbon; DOC, dissolved organic C; MBC, microbial biomass C; MBN, microbial biomass nitrogen.

**Supplementary TABLE S3** Pairwise PERMANOVA results for soil bacterial community composition among treatments.

| Comparison | Df | *R*^2^ | *F* | *p* |
| --- | --- | --- | --- | --- |
| Control vs. B300 | 1 | 0.65 | 11.0 | 0.031 |
| Control vs. B500 | 1 | 0.36 | 3.37 | 0.027 |
| Control vs. B900 | 1 | 0.48 | 5.46 | 0.030 |
| Control vs. Charcoal | 1 | 0.50 | 6.03 | 0.027 |
| B300 vs. B500 | 1 | 0.53 | 6.89 | 0.029 |
| B300 vs. B900 | 1 | 0.63 | 10.4 | 0.030 |
| B300 vs. Charcoal | 1 | 0.63 | 10.2 | 0.028 |
| B500 vs. B900 | 1 | 0.37 | 3.45 | 0.028 |
| B500 vs. Charcoal | 1 | 0.36 | 3.33 | 0.032 |
| B900 vs. Charcoal | 1 | 0.39 | 3.81 | 0.029 |

B300, B500, and B900 denote biochars pyrolyzed at 300, 500, and 900 ℃, respectively.

**Supplementary TABLE S4** Relative abundance (%) of dominant soil bacterial phylum under different char addition treatments (mean ± SE, n=4).

| Treatment | Control | B300 | B500 | B900 | Charcoal |
| --- | --- | --- | --- | --- | --- |
| Acidobacteriota | 34.6 ± 0.31 a | 27.8 ± 0.42 d | 32.9 ± 0.47 c | 33.5 ± 0.55 b | 35.0 ± 0.48 a |
| Proteobacteria | 27.7 ± 0.25 c | 41.2 ± 0.35 a | 30.0 ± 0.29 b | 26.6 ± 0.37 c | 27.2 ± 0.49 c |
| Chloroflexi | 8.03 ± 0.16 a | 5.04 ± 0.09 d | 7.12 ± 0.13 b | 6.61 ± 0.09 c | 7.61 ± 0.10 a |
| Verrucomicrobiota | 6.92 ± 0.15 b | 5.74 ± 0.16 c | 6.73 ± 0.16 b | 8.45 ± 0.44 a | 6.99 ± 0.28 b |
| Gemmatimonadota | 0.92 ± 0.03 a | 0.62 ± 0.03 b | 0.90 ± 0.03 a | 0.95 ± 0.05 a | 0.85 ± 0.02 a |
| Armatimonadota | 0.61 ± 0.03 b | 0.50 ± 0.01 c | 0.73 ± 0.02 a | 0.66 ± 0.02 ab | 0.63 ± 0.02 ab |
| Elusimicrobiota | 0.53 ± 0.03 d | 0.58 ± 0.03 cd | 0.66 ± 0.04 bc | 0.88 ± 0.08 a | 0.75 ± 0.05 b |
| Bdellovibrionota | 0.48 ± 0.03 b | 0.72 ± 0.04 a | 0.47 ± 0.04 b | 0.78 ± 0.05 a | 0.47 ± 0.02 b |
| Methylomirabilota | 0.35 ± 0.03 a | 0.23 ± 0.01 c | 0.29 ± 0.01 b | 0.29 ± 0.01 ab | 0.23 ± 0.01 c |
| Cyanobacteria | 0.21 ± 0.01 c | 0.40 ± 0.02 a | 0.29 ± 0.01 b | 0.29 ± 0.02 b | 0.25 ± 0.01 bc |
| Dependentiae | 0.08 ± 0.01 a | 0.05 ± 0.01 b | 0.09 ± 0.01 a | 0.09 ± 0.01 a | 0.05 ± 0.01 b |

Different lowercase letters within the same row indicate significant differences among treatments (*p* < 0.05). B300, B500, and B900 denote biochars pyrolyzed at 300, 500, and 900 ℃, respectively.

**Supplementary TABLE S5** Relative abundance (%) of dominant soil bacterial genus under different char addition treatments (mean ± SE, n=4).

| Treatment | Control | B300 | B500 | B900 | Charcoal |
| --- | --- | --- | --- | --- | --- |
| Candidatus_U | 3.78 ± 0.10 b | 2.88 ± 0.07 c | 3.60 ± 0.15 b | 4.67 ± 0.29 a | 3.87 ± 0.18 b |
| Bradyrhizobium | 1.82 ± 0.04 d | 3.47 ± 0.06 a | 2.11 ± 0.06 b | 1.93 ± 0.08 c | 1.60 ± 0.08 e |
| Bryobacter | 1.73 ± 0.05 ab | 1.42 ± 0.03 c | 1.65 ± 0.06 b | 1.88 ± 0.06 a | 1.74 ± 0.06 ab |
| Candidatus_X | 1.42 ± 0.04 bc | 1.26 ± 0.09 c | 1.50 ± 0.07 ab | 1.66 ± 0.09 a | 1.39 ± 0.05 bc |
| Burkholderia | 0.82 ± 0.04 c | 6.10 ± 0.22 a | 1.42 ± 0.13 b | 1.05 ± 0.01 bc | 1.44 ± 0.18 b |
| Occallatibacter | 0.67 ± 0.03 a | 0.52 ± 0.03 b | 0.66 ± 0.03 a | 0.63 ± 0.02 a | 0.68 ± 0.04 a |
| Subgroup_5 | 0.67 ± 0.03 b | 0.50 ± 0.01 c | 0.67 ± 0.01 b | 0.80 ± 0.04 a | 0.68 ± 0.04 b |
| Edaphobacter | 0.40 ± 0.02 a | 0.32 ± 0.02 a | 0.37 ± 0.02 a | 0.37 ± 0.02 a | 0.37 ± 0.03 a |
| Granulicella | 0.36 ± 0.01 a | 0.27 ± 0.02 b | 0.34 ± 0.03 ab | 0.39 ± 0.01 a | 0.38 ± 0.04 a |
| Lineage_IIa | 0.31 ± 0.02 cd | 0.29 ± 0.01 d | 0.38 ± 0.03 bc | 0.61 ± 0.03 a | 0.46 ± 0.02 b |
| Gemmatimonas | 0.28 ± 0.02 b | 0.19 ± 0.02 c | 0.34 ± 0.01 ab | 0.37 ± 0.03 a | 0.36 ± 0.02 a |
| Phenylobacterium | 0.23 ± 0.01 b | 0.38 ± 0.02 a | 0.23 ± 0.02 b | 0.26 ± 0.01 b | 0.23 ± 0.00 b |
| Subgroup_13 | 0.24 ± 0.02 a | 0.14 ± 0.01 b | 0.23 ± 0.01 a | 0.21 ± 0.03 a | 0.22 ± 0.01 a |

Different lowercase letters within the same row indicate significant differences among treatments (*p* < 0.05). B300, B500, and B900 denote biochars pyrolyzed at 300, 500, and 900 ℃, respectively.
